# Interstitial cystitis with Hunner's ulcers managed with Jatyadi Taila Uttarabasti - A case series

**DOI:** 10.1016/j.jaim.2026.101358

**Published:** 2026-07-24

**Authors:** Kaushal Patel, Manish Patel, Chintan Bhatt

**Affiliations:** a, Mahakali Ayurveda Hospital and Panchakarma Centre, Halol, India; b Department of Kayachikitsa, J. S. Ayurveda College, Nadiad, India; c Department of Panchakarma, J. S. Ayurveda College, Nadiad, India

**Keywords:** Interstitial cystitis (IC) treatment, Bladder pain syndrome treatment (BPS), Hunner's ulcer, *Uttarbasti* treatment (intravesical instillation), Panchakarma treatment in interstitial cystitis, Ayurveda, Case series

## Abstract

Interstitial cystitis (IC) with Hunner's ulcers is a chronic inflammatory condition often resistant to conventional therapies, which frequently offer only temporary relief or carry risks of bladder scarring. This report describes the clinical outcomes of *Jatyadi Taila Uttarabasti* (intravesical instillation) combined with *Virechana Karma* (purgation) and oral formulations—in managing severe IC.

Two patients with chronic IC and Hunner's ulcers, non-responsive to conventional treatments, received individualized Ayurvedic treatment at the hospital. Clinical progress was documented using the Visual Analogue Scale (VAS) for pain, the King's Health Questionnaire (KHQ), and the IC-Quality of Life (IC-QoL) score, with a follow-up period of two years.

Both patients demonstrated significant clinical improvement. Pain scores reduced from severe to near-zero, and urinary frequency and nocturia were normalized. Quality-of-life metrics showed substantial enhancement, with IC-QoL scores reflecting near-complete symptom resolution. Notably, these results remained stable throughout the two-year follow-up period. Patient 2 opted for a planned maintenance course in late 2024 to ensure clinical stability, despite the resolution of primary symptoms.

This case series suggests that Ayurvedic management, particularly the use of *Jatyadi Taila Uttarabasti* for its wound-healing and anti-inflammatory properties, provides a cost-effective, non-invasive, and long-lasting alternative for severe IC and Hunner's ulcers.

## 1 Introduction

### 1.1 Background of interstitial cystitis (IC) and clinical challenge

IC also known as Bladder Pain Syndrome (BPS), is a chronic and debilitating inflammatory disorder of the bladder, presenting a complex clinical challenge due to its unclear etiology. It Affects an estimated 3-8 million women and 1-4 million men in the United States [1]. It is characterized by persistent pelvic pain, urinary urgency, and increased frequency. 5% of them have Hunner's ulcer. IC/BPS patients with Hunner ulcers usually have higher symptom scores, increased urinary frequency, more nocturia episodes, smaller voided volumes, and a reduced bladder capacity with hydrodistension than IC/BPS patients without Hunner ulcers [2]. Hunner ulcers exacerbate symptoms and complicate management. The pathogenesis is linked to a compromised glycosaminoglycan (GAG) layer, allowing urinary solutes to penetrate the urothelium and trigger chronic inflammation.

### 1.2 Limitations of conventional treatments

Conventional treatments, such as nonsteroidal anti-inflammatory drugs (NSAIDs), tricyclic antidepressants, and antihistamines, often provide only temporary symptomatic relief [3]. However, they fail to address the root cause of the condition, and comes with considerable side effects, including gastrointestinal issues, renal impairment, and an increased risk of bleeding [4]. Surgical interventions like bladder hydrodistention and ulcer cauterization carry additional risks such as bladder scarring and reduced bladder capacity, which can further impact patients' well-being [5].

Conventional intravesical drug delivery aims to manage these symptoms by delivering high concentrations of medication directly to the bladder. However, there are significant limitations to this approach. Key challenges include the short duration of action of intravesically administered drugs, as they are often rapidly eliminated with the first voiding. Additionally, the inherent impermeability of the bladder restricts the deep penetration of therapeutic agents into the urothelial layers, reducing their efficacy [6].

### 1.3 Role of liposomes in intravesical therapy

To address the limited permeability of the bladder wall, intravesical delivery methods have evolved to modulate the release and absorption characteristics of instilled drugs, often through the use of novel carriers such as intravesical liposomes. Lipid solubility plays a crucial role in drug transport across biological membranes. Drugs with high lipophilicity will easily penetrate the urothelium [6]. Recent advances in the intravesical treatment of interstitial cystitis include the use of intravesical liposomes. Beyond their application in urology, liposomes have demonstrated wound-healing properties by forming a moisture-retaining film over wounds without triggering inflammatory responses [7].

### 1.4 Ayurvedic rationale: Selection of Jatyadi Taila for mucosal repair

There is a critical need for cost-effective, non-invasive therapies that provide sustained mucosal healing. In Ayurveda, lipid-based formulations—*Taila* (medicated oils) and *Ghrita* (medicated ghee)—are utilized to enhance the bioavailability and retention of phytoconstituents in target tissues. While numerous Ayurvedic formulations target urogenital disorders, Jatyadi Taila was specifically selected for clinical management due to its classical indication for *Dushta Vrana* (chronic, non-healing ulcers). Given that Hunner's ulcers represent a focal failure of the bladder's damage-repair process, the potent wound-healing (*Vrana Ropana*) and anti-inflammatory (*Shothahara*) properties of *Jati*, *Nimba*, and *Suddha Tuttha* (purified copper sulphate) make it an ideal candidate for targeted intravesical therapy [8].

Mechanistically, *Jatyadi Taila* serves as a natural lipophilic medium that mimics the protective function of the compromised glycosaminoglycan (GAG) layer. Administered via Uttarabasti (intravesical instillation), the oil's lipid-based structure allows it to adhere to the lipid-rich urothelium, ensuring longer residence time and deeper penetration of phytoconstituents than aqueous solutions. Acharya Sushruta has mentioned in *Uttarasthana* while describing treatment for *Mutrakriccha* that after *Snehapana* (Structured oral administration of medicated ghee)*, Abhyanga* (30-min full-body massage)*, Svedana* (steam therapy) *Virechana* (therapeutic purgation) has to be performed after proper *Virechana Karma Uttarbasti* has to be given [9].

Also emerging evidence supports a gut-bladder axis in the pathogenesis of interstitial cystitis (IC), where specific gut microbiota taxa are implicated in systemic and local inflammatory responses. Ayurvedic interventions such as *Dashamoola Niruha Basti* (Medicated decoction enema) and *Bala Taila Matra Basti* support gut health and modulate inflammation and may play a dual role in managing IC by influencing both gut microbial composition and bladder inflammation [10].

This case series aims to contribute to the existing knowledge of IC management by documenting the clinical observations of Ayurvedic treatment in providing sustained relief and promoting bladder mucosal healing. It highlights a treatment approach that focuses not only on symptomatic relief but also on addressing the underlying pathology of the condition. The findings of this cases could expand the therapeutic options available to clinicians managing IC and BPS, offering insights into integrative strategies that can enhance patient care in this challenging condition.

And also to explore *Jatyadi Taila* as a natural and cost-effective alternative to synthetic liposomal formulations. It also aims to document its potential for prolonged therapeutic benefits by healing bladder mucosa and reducing inflammation.

## 2 Case Presentation

### 2.1 Case Presentation: patient 1

A 62-year-old female housewife presented to the OPD of PD Patel Ayurveda Hospital, Nadiad on 6 December 2022 with chronic Interstitial Cystitis (IC). The patient also had a comorbid 12-year history of Diabetes Mellitus. Her condition was refractory to conventional allopathic treatments, including previous fulguration of Hunner's ulcers.

#### 2.1.1 Clinical findings

Upon presentation to the outpatient department, the patient reported a debilitating symptom complex that had persisted for 11 years. The primary clinical findings included:

• **Urgency and Voiding Character:** The patient described an intense urgency where she was voiding every 5 minutes, often passing only drops of urine.
• **Chronic Pelvic Pain:** The patient experienced persistent dysuria and severe, forceful pain localized to the urethral, anal, and vaginal regions.
• **Urinary Frequency:** The patient exhibited extreme urinary frequency, voiding approximately 20–25 times per day.
• **Impact of Infection:** These symptoms were exacerbated by a history of repeated urinary tract infections (UTIs) occurring between 2019 and 2022.
• **Treatment Refractoriness:** The patient demonstrated a lack of symptomatic response to both prolonged antibiotic therapy and previous surgical fulguration of Hunner's ulcers.
• **Quality of Life Impact:** The constant urge to urinate and the associated pain resulted in significant sleep disturbances and the inability to perform daily social or household activities.

#### 2.1.2 Diagnostic assessment

Baseline evaluations confirmed severe IC with Hunner's ulcers:

• Imaging**:** A pre-intervention USG (March 2022) revealed a pre-void volume of 110 cc and a post-void residual urine (PVRU) of 55 cc.
• Cystoscopy & Histopathology**:** Diagnostic cystoscopy (December 2018) confirmed three Hunner's ulcers with a bladder capacity of 200 ml. Biopsy results indicated acute and chronic inflammation with ulceration, showing aggregates of lymphocytes and plasma cells in the lamina propria.
• Microbiology: Documented recurrent urinary tract infections (UTIs) with various organisms between 2019 and 2022 as detailed in Supplementary File, requiring prolonged antibiotic therapy with minimal relief.

#### 2.1.3 Timeline

The patient's disease progression and the timeline of Ayurvedic interventions are summarized in Table 1; notably, despite the fulguration of Hunner's ulcers in 2018, the patient's symptoms remained unresolved until the commencement of the current Ayurvedic treatment plan involving *Virechana Karma* and *Jatyadi Taila Uttarabasti*.

**Table 1:** Patient 1 timeline.

Date	Event/Procedure	Bladder Capacity/Metrics
2013	Onset of persistent pain in the urethra, anus, and vaginal region; Pain intensified during urination, with urinary frequency of 15–20 times/day and nocturia 8–10 times/night.	—
02-Oct-2018	Ultrasound showed a mildly distended bladder with a diffusely thickened wall (7 mm pre-void, 10 mm post-void).	Pre-void: 37 cc Post-void: 4 cc
06-Dec-2018	Symptoms intensified with severe pain in the lower abdomen and perineum, dysuria, and increased urinary frequency., which had affected daily activities.	—
15-Dec-2018	Cystoscopy with hydrodistention and fulguration of three Hunner's ulcers was performed; biopsy confirmed IC with ulceration.	200 ml (during hydrodistention)
17-Dec-2018	Histopathology confirmed acute and chronic inflammation with ulceration.	—
25-Jul-2020	Ultrasound showed moderately distended bladder with diffusely thickened wall (6 mm pre-void, 9 mm post-void).	Pre-void: 143 cc 1st Post-void: 45 cc 2nd Post-void: 11 cc
06-Dec-2022	Hospital Admission: Symptoms: Severe urinary symptoms persisted despite prolonged antibiotic treatment and fulguration of Hunner's ulcers. Patient experienced urination every 5 minutes, often by drops, with severe, forceful pain throughout the day, greatly impacting personal, social life, and overall quality of life before admission to the hospital. Admitted to PD Patel Ayurveda Hospital from 6 December 2022 to 5 January 2023 for treatment with Uttarabasti., Upon discharge from the hospital she gets significant relief in her symptoms and advised her to continue oral treatment at home and come regularly for followup on every 28th day.	—
05-Jan-2023	Discharged with significant symptomatic relief; advised to continue oral Ayurvedic medications.	—
21-Apr-2023	Event: Patient experienced a stroke and underwent coronary artery bypass graft (CABG) on the same day. As a result, she was unable to continue oral Ayurvedic medications for 2 months.	
01-Sep-2023 to 29-Sep-2023	Patient came for followup after 6months and was hospitalized again as she discontinued oral medications since long, and treated with Uttarabasti. Post-treatment, she resumed Ayurvedic oral medications.	
06-Feb-2023	Imaging: Ultrasound KUB showed moderately distended bladder with multiple well-defined, round, non-dependent echogenic soft tissue nodules along the dome of the bladder (largest nodule: 22 × 13 mm; combined measurement: 54x23 × 17 mm), suggestive of possible fat-containing nodules.	—
06-Apr-2024	Imaging: Ultrasound showed bladder wall diffusely thickened with mobile debris and small clots.	Pre-void: 160 cc Post-void: 10 cc
28-Oct-2024	Final Follow-up: Long-term relief achieved; no pain reported, frequency reduced to 8–9 times/day,with the ability to hold urine for 2-2.5 hours (previously only 5 minutes). and quality of life fully restored.	

### 2.2 Case Presentation: patient 2

A 53-year-old male farmer presented to the OPD on 8 March 2024, with a three-year history of debilitating urinary symptoms. The patient had a history of seeking care at multiple hospitals, where he was managed with NSAIDs and analgesics without symptomatic relief. Although surgical intervention for Hunner's ulcers had been recommended by his urologist, the patient opted for Ayurvedic management.

#### 2.2.1 Clinical findings

At the time of presentation, the patient reported a severe symptom complex since past 3years characterized by:

• **Localized Pain:** Severe, spasmodic suprapubic pain and pain at the glans penis that intensified during micturition.
• **Urinary Frequency and Nocturia:** Diurnal frequency was recorded at 10–15 times per day, with extreme nocturia of 15–20 episodes per night.
• **Voiding Character:** The patient experienced persistent dribbling of urine.
• **Functional Impact:** These symptoms were refractory to conventional allopathic medical management.

#### 2.2.2 Clinical timeline

• The patient's disease progression and the timeline of Ayurvedic interventions are summarized in Table 2.

**Table 2:** Patient 2 timeline.

Date	Event/Procedure	Clinical Details	Bladder Capacity/Metrics
2020	Onset of Symptoms	Initial onset of pain in the glans penis and increased urinary frequency.	—
18-Jun-2022	Ultrasound of Urinary System	Revealed mild bladder wall thickening and a left renal cortical cyst.	Pre-void: 90 ml; Post-void: Nil
30-Jun-2022	Uroflowmetry	Baseline assessment of voiding dysfunction.	Voided: 77.6 ml; Maximum flow rate: 4.7 ml/sec Average flow rate: 2.5 ml/sec
07-Oct-2022	Diagnostic Cystoscopy	Confirmed IC with Hunner's ulcers; multiple dilated vessels and reddish mucosal patches observed. **Patient refused recommended fulguration**.	200 ml (during hydrodistention)
15-Dec-2022	Uroflowmetry	Follow-up assessment showing persistent low flow rates.	Voided: 129 ml; Maximum flow rate: 4 ml/sec Average flow rate: 1.3 ml/sec
18-Mar-2024 to 19-Apr-2024	First Admission to PD Patel Ayurveda Hospital	Admitted for intensive Ayurvedic management as per the treatment plan in Table 3.	Significant symptomatic relief achieved.
29-Apr-2024	Abdominal and Pelvic Ultrasound	Imaging showed mild wall thickening with minimal trabeculations; consistent with clinical improvement in pain and frequency.	Pre-void: 114 cc; Post-void: 10 cc
09-Nov-2024 to 09-Dec-2024	Second Admission to PD Patel Ayurveda Hospital	Readmitted for a second course of therapy (see Table 3) at the patient's request.	Complete resolution of pain and nocturia; avoided invasive surgery.

#### 2.2.3 Diagnostic assessment

Diagnostic evaluations conducted prior to the current admission confirmed severe IC and significant voiding dysfunction:

• **Cystourethroscopy (07 October 2022):** Findings revealed multiple dilated vessels across the bladder mucosa and characteristic reddish patches (Hunner's ulcers) on both lateral walls.
• **Hydrodistention:** Performed to a capacity of 200 ml for 5 minutes during the cystoscopic procedure.
• **Uroflowmetry (15 December 2022):** Results indicated severe dysfunction with a voided volume of 29 ml and a maximum flow rate of only 4 ml/sec. The flow time was 12 seconds with an interval time of 5 seconds.

## 3 Therapeutic Intervention

The treatment plan during hospitalization is given in Table 3. We have started with *Snehapana* (Internal administration of ghee) using *Gokshuradi Ghrita*, administered in an increasing dosage from 40 to 120 ml twice a day with warm water over 3 to 7 days, adjusted based on each patient's *Kostha* (bowel nature) and *Agni* (digestive fire). After completing *Snehapana*, *Sarvanga Abhyanga* with *Narayana Taila* was performed. After *Abhyanga, Sarvanga Bashpa Swedana* using *Nirgundi Patra* is performed for 10 minutes every day, excluding on the days of *Samsarjanakarma* (post-purification diet regimen). *Virechana Karma* is administered on the third day of *Abhyanga* and *Swedana*, using *Eranda Sneha* combined with *Dindayal Churna*, in dosages adjusted to the patient's *Kostha*. *Samsarjanakarma* follows for 2–3 days, depending on the degree of purification achieved. Oral medications include *Varunadi Kwath* (40 ml morning and evening), *Rasayana Churna* (3 g twice daily), and *Gokshuradi Guggulu* (1 g three times daily), each continued for rest 3 weeks of hospitalization. *Avagaha Swedana* (sitz bath) with *Nimba Patra*, is performed twice daily in the morning and just before *Uttarabasti karma*. *Niruha Basti* (Medicated enema) with *Dashamoola Kwatha* (320 ml in the morning before food on alternate days) and *Matra Basti* (Oil based enema) with *Bala Taila* (40 ml in the evening after food), both administered on days alternate to each other for the rest 3 weeks of hospitalization. *Uttara Basti* with *Jatyadi Taila*, administered 20 ml daily in the morning, following *Abhyanga*, *Swedana*, and *Avagaha Swedana*, for 3 weeks.

**Table 3:** Treatment plan.

Sr. No	Treatment	Medicine	Dosage.	Duration
1	*Snehapana*	*Gokshuradi Ghrita*	40ml - 120ml (In increasing dosage everyday)	3-7 days according to the *Koshtha* & *Agni* of the patients.
2	*Sarvang Abhyanga* [13]	*Narayana Taila*	30mins	After completing *Snehapana* for 3days, and everyday after *Samsarjanakarma.*
3	*Sarvang Bashpa Swedana* [13]	*Nirgundi Patra*	10mins	After completing *Snehapana* for 3days, and everyday after *Samsarjanakarma.*
4	*Virechana Karma* [14]	*Eranda Sneha + Dindayal Churna.*	40-60 ml+ 5-6g. (depending upon *Kostha*)	On the 3rd day of *Abhyanga* and *Svedana*.
5	*Samsarjan Karma*			2-3 days as per S*huddhi*
6	Oral medicaments	*Varunadi Kwath.* [15]	40ml-0-40ml Empty stomach (morning and evening)	Rest 3 weeks of hospitalization and continued on OPD basis after discharge from the hospital.
7		*Rasayana churna.*	3g × 2times With warm water	
8		*Gokshuradi* *Guggulu* [16]	1g × 3times With warm water	
9	*Avagaha Swedana* [17]	*Nimba Patra*	2 times, in the morning and just before *Uttarabasti*.	3 weeks
10	*Niruha Basti*	*Dashamoola* *Kwatha* [18]	320ml in the morning before food 01:00 p.m.	3 weeks during hospitalization (On Alternate days)
11	*Matra Basti*	*Bala Taila* [19]	40 ml in the evening after food 07:00 p.m.	
12	*Uttara Basti*	*Jatyadi Taila*	20ml	3 weeks (Everyday in the morning 8:00 a.m. after *Abhyanga, Svedana and Avagaha Swedana)*

### 3.1 Preparation of *Jatyadi Taila*

*Jatyadi Taila* was prepared according to classical *Taila Paka Vidhi* as described in the *Sharangdhara Samhita* [11].

To ensure safety for intravesical administration, the following quality and sterility protocols were followed:

• **Manufacturing**: The oil was manufactured at Sundar Ayurveda Pharmacy, a GMP-certified facility within the institute, ensuring batch-to-batch consistency and adherence to standardized manufacturing practices.
• **Sterilization**: The final oil was subjected to autoclaving to ensure a sterile and pyrogen-free medium suitable for intravesical administration.
• **Quality Control**: Each batch underwent mandatory microbiological testing to confirm the absence of aerobic and anaerobic pathogens (e.g., *E. coli*, *S. aureus*, and *Pseudomonas*) before clinical use.
• The oil was manufactured at Sundar Ayurveda Pharmacy, a GMP-certified facility within the institute, ensuring batch-to-batch consistency and adherence to standardized manufacturing practices.
• **Sterilization**: The final oil was subjected to double filtration (including a 0.22-μm membrane filter) and autoclaving to ensure it was sterile and pyrogen-free.
• **Quality Control**: Each batch underwent mandatory microbiological testing to confirm the absence of aerobic and anaerobic pathogens (e.g., *E. coli*, *S. aureus*, and *Pseudomonas*) before clinical use.

### 3.2 *Uttarabasti* procedure

The *Uttarabasti* procedure utilized 20 ml of sterile *Jatyadi Taila* per session. To ensure patient safety and procedural success, the following clinical protocol was implemented:

Aseptic Precautions and Administration**:** Strict surgical asepsis was maintained throughout the entire procedure, extending beyond the preparation phase. Preparatory measures included shaving the pubic hair and a 15-min *Avagaha Sveda* (sitz bath) using a decoction of *Nimba Patra* (*Azadirachta indica*). After the patient emptied their bladder and given a supine position, the pubic region and genitalia were thoroughly cleansed with an antiseptic solution. The procedural field was maintained by covering the area with a sterile green drape secured with towel clips.

The clinician utilized sterile gloves and a "no-touch" technique to handle an autoclaved rubber catheter of appropriate size. The catheter, attached to a disposable syringe prefilled with sterilized, warmed *Jatyadi Taila*, was lubricated with sterile oil and gently inserted into the urethra to instill the medication into the bladder. Following instillation, the catheter was carefully withdrawn, and the urethral orifice was covered with sterile cotton and gauze.

**Monitoring and Complication Management:** Patients were monitored continuously throughout the 30-min dwell time for any adverse reactions, including:

• **Immediate Responses:** Monitoring for vasovagal syncope, acute bladder spasms, or localized burning sensations.
• **Potential Complications:** Assessment for secondary urinary tract infections (UTIs), hematuria, or urethral trauma.
• **Tolerability**: Despite the presence of *Suddha Tuttha* in the formulation, no complications or discomfort were reported by the patients across the 30-day treatment regimen.

The oil was retained in the bladder for a minimum dwell time of 30 minutes, allowing maximal contact with the bladder mucosa to enhance therapeutic absorption. After this period, the patient was permitted to void naturally. The procedure was performed once daily for 30 consecutive days, ensuring a consistent and thorough treatment regimen.

## 4 Follow-up and Outcomes

The clinical outcomes of these cases underscore the significant impact of Ayurvedic intervention on the quality of life for patients suffering from severe interstitial cystitis with Hunner's ulcers. Beyond the objective numerical improvements, both patients experienced relief from debilitating symptoms that had previously severely restricted their ability to engage in daily social and occupational activities. Overall, both subjects achieved over an 80% reduction in standardized symptom scores and near-complete to complete pain resolution, which remained stable throughout the two year follow-up period. Detailed metrics are summarized in Table 4.

**Table 4:** Assessment before and after treatment.

Assessment Tool	Patient 1 (Baseline)	Patient 1 (Post-Tx)	Patient 2 (Baseline)	Patient 2 (Post-Tx)
VAS Scale for Pain	9/10	0/10	6/10	1/10
ICSI Score	20	03	14	4
ICPI Score	16	01	11	3
PUF Symptom Scale	29	03	24	10
KHQ (Impact %)	95%	15%	60%	25%
PCS Score	52	6	25	15
IC-QoL Score	40	02	34	01

### 4.1 Patient 1 Outcomes

Patient 1 achieved a profound clinical response, characterized by following matrics and normalization of urinary frequency and complete cessation of pelvic pain.

• **Pain Resolution:** Chronic pelvic pain was entirely resolved, with the Visual Analog Scale (VAS) score decreasing from 9/10 to 0/10 (100% improvement).
• **Standardized Symptom Indices:** Remarkable declines were observed in the Interstitial Cystitis Symptom Index (ICSI) and Problem Index (ICPI), which improved by 85% and 93.8%, respectively. The Pelvic Pain and Urgency/Frequency (PUF) score dropped by 89.7%, reflecting a significant reduction in voiding distress.
• **Quality of Life (QoL) and Psychological Impact:** The King's Health Questionnaire (KHQ) impact score was reduced by 84.2%, while the IC-Quality of Life (IC-QoL) score improved by 95%. Pain catastrophizing behaviors also decreased by 88.5%.
• **Microbiological Clearance:** Following the resolution of chronic, multi-organism recurrent infections that persisted for three years (Table 4), the patient achieved 100% microbiological clearance. No pathogens were detected at the two-year follow-up (August 2024), and the patient remained free of antibiotic therapy.
• **Long-term Follow-up:** A negative urine culture on 30-Aug-2024 and a final follow-up on 28-Oct-2024 documented that the patient remained asymptomatic without medication for 24 months, with an improved urine hold time of 2–2.5 hours.

### 4.2 Patient 2 Outcomes

Patient 2 demonstrated substantial clinical and objective improvements, particularly regarding severe nocturia and chronic voiding dysfunction. Overall, the patient achieved a significant reduction in symptom burden, allowing for a complete return to occupational activities.

• **Uroflowmetry and Voiding Parameters:** Post-treatment objective assessment revealed a significant increase in voided volume, which improved from a baseline of 29 ml to 145 ml. The maximum flow rate (Q_*max*_) concurrently improved from 4 ml/sec to 11.2 ml/sec, reflecting a restoration of physiological bladder function. Diurnal urinary frequency and nocturia episodes were successfully reduced to physiological ranges.
• **Pain Reduction:** The Visual Analogue Scale (VAS) score for micturition-associated pain decreased from 6/10 to 1/10, representing an 83.3% improvement.
• **Standardized Symptom Indices:** The Interstitial Cystitis Symptom Index (ICSI) and Problem Index (ICPI) improved by 71.4% and 72.7%, respectively. The Pelvic Pain and Urgency/Frequency (PUF) symptom scale demonstrated a 58.3% reduction in overall clinical burden.
• **Functional and Psychosocial Recovery:** Quality-of-life metrics showed a nearly complete restoration, with a 97.1% improvement in the IC-QoL score and a 58.3% reduction in the King's Health Questionnaire (KHQ) impact score. This enabled the patient to resume daily social and farming duties without the need for previously recommended surgical intervention.
• **Clinical Stability and Maintenance:** While a second admission was recorded from 9 November to 9 December 2024, this was a planned, patient-requested maintenance course aimed at ensuring long-term stability rather than a response to symptom recurrence. The patient remained asymptomatic between the two treatment courses.
• **Objective Imaging:** An abdominal and pelvic ultrasound conducted on 29 April 2024 documented mild wall thickening with minimal trabeculations, reflecting significant mucosal recovery and a reduction in chronic inflammatory markers compared to baseline diagnostic findings.

## 5 Discussion

The pathophysiology of Interstitial Cystitis (IC) involves a multifactorial cascade culminating in the dysfunction of the glycosaminoglycan (GAG) layer and subsequent submucosal inflammation. While modern intravesical therapies aim to repair this barrier, they often lack direct anti-inflammatory properties and suffer from short drug retention times, frequently failing to achieve long-term remission [7]. Ayurvedic principles offer a complementary perspective by correlating IC with *Mutrakrichra* (difficulty in urination), emphasizing a holistic approach that targets both localized bladder pathology and systemic imbalances.

### 5.1 Mechanistic insights: Jatyadi Taila and lipid-based delivery

The clinical efficacy of *Jatyadi Taila* in this case series aligns with modern research into intravesical lipid-based drug delivery systems, which suggests that lipophilic mediums with structural similarities to natural cell membranes demonstrate superior outcomes in reducing bladder contraction frequency and improving symptoms [12]. As a lipophilic medium, the oil-based composition of *Jatyadi Taila* allows it to adhere to the lipid-rich urothelium, forming a sustained protective coating. This mechanism increases the residence time of bioactive phytoconstituents at the site of inflammation, providing a mechanical barrier that likely supplements the compromised glycosaminoglycan (GAG) layer. By shielding the mucosa from irritation by urinary solutes and fostering a therapeutic environment conducive to the healing of Hunner's ulcers, this approach addresses the limitations of standard aqueous treatments.

### 5.2 Biofilm disruption and gut-bladder axis

The resolution of recurrent urinary tract infections (UTIs) observed in this series suggests a potential, though theoretical, impact on the biological environment of the bladder. It is hypothesized that *Jatyadi Taila* may minimize conditions favoring biofilm persistence by strengthening natural mucosal defenses and reducing local inflammation. While further laboratory assays are required to confirm biofilm disruption, the clinical clearance of chronic organisms suggests a less favorable environment for bacterial persistence. Furthermore, the concurrent use of *Dashamoola Niruha Basti* suggests a synergistic effect via the "gut-bladder axis." Modulating the gut microbiota through systemic Ayurvedic interventions may influence immune signaling pathways, thereby augmenting the local effects of *Uttarabasti* and reducing systemic inflammatory triggers.

### 5.3 Case series limitations

Despite the significant improvements in patient-reported outcomes (PROs) measured via validated tools such as the IC-QoL and VAS, this case series has several inherent limitations. First, the small cohort size (n = 2) restricts the generalizability of these results to the broader Interstitial Cystitis and Bladder Pain Syndrome (IC/BPS) population. Second, as an uncontrolled case series, the findings are based on clinical observations without a parallel control group. While the long-standing, refractory nature of these cases—which remained unresolved despite multiple prior allopathic interventions—suggests a robust therapeutic response, larger comparative trials are necessary to confirm these observations across a wider demographic.

## 6 Patient perspective

Patient 1 (Recorded on: 28 October 2024) "Before starting Ayurvedic treatment, I had severe pain and had to pass urine every few minutes, which made it impossible to sleep or carry out my daily activities. Despite previous surgeries and prolonged medications, I did not experience lasting relief. After undergoing the Ayurvedic treatment, my pain gradually disappeared, I could hold urine for much longer, and I was able to return to my normal routine. I am satisfied with the treatment and the sustained improvement in my quality of life."

Patient 2 (Recorded on: 09 December 2024) "My urinary pain and frequent night-time urination had greatly affected my work and daily life. I had been advised to undergo surgery, but I chose Ayurvedic treatment instead. After treatment, my symptoms improved significantly, and I was able to resume farming and my normal activities without surgery. I am pleased with the outcome and grateful for the sustained relief I have experienced."

## 7 Conclusion

This case series demonstrates that an integrated Ayurvedic approach, specifically the use of *Jatyadi Taila Uttarabasti* alongside systemic purification, offers a potentially promising alternative for the management of refractory Interstitial Cystitis with Hunner's ulcers. Both patients achieved significant and stable relief from pain and urinary frequency, which was maintained over a two-year follow-up period. By utilizing the lipid-based properties of traditional medicated oils to provide prolonged mucosal contact and anti-inflammatory action, this treatment offers a potentially appears promising alternative to conventional intravesical therapies and invasive procedures. While these findings are encouraging, larger randomized controlled trials are required to validate these results and to further explore the integration of Ayurvedic treatment into mainstream IC management strategies.

## Informed consent

Written permission for the publication of these case reports were obtained from the patients.

## Author's declarations

The authors certify that they have obtained all appropriate patient consent forms. In the form, the patients have given consent for images and other clinical information to be reported in the journal. The patients understands that their name and initials will not be published and due efforts will be made to conceal their identity.

## Author contributions

All authors contributed to the acquisition of data, analysis, and interpretation of results. Each author reviewed and approved the final version of the manuscript for submission. Additionally, the corresponding author ensured that all aspects of the work were appropriately addressed and that any questions related to the accuracy or integrity of the work were resolved.

## Declaration of generative AI in scientific writing

During the preparation of this work, the author (corresponding author) declares that no tools, services, or AI technologies were utilized in the writing process. Only basic tools, such as the Grammarly app for grammar checking, were employed. The author has no other disclosures to make regarding the use of generative AI in this manuscript.

## Funding sources

None.

## Conflict of interest

There is no conflict of interest.
